# Effects of Interleukin-17A on Osteogenic Differentiation of Isolated Human Mesenchymal Stem Cells

**DOI:** 10.3389/fimmu.2014.00425

**Published:** 2014-09-02

**Authors:** Bilal Osta, Fabien Lavocat, Assia Eljaafari, Pierre Miossec

**Affiliations:** ^1^Immunogenomics and Inflammation Research Unit EA 4130, Department of Clinical Immunology and Rheumatology, Edouard Herriot Hospital, University of Lyon 1, Lyon, France

**Keywords:** mesenchymal stem cell, IL-17A, TNF-α, rheumatoid arthritis, ankylosing spondylitis

## Abstract

**Objectives:** Rheumatoid arthritis (RA) is characterized by defective bone repair and excessive destruction and ankylosing spondylitis (AS) by increased ectopic bone formation with syndesmophytes. Since TNF-α and IL-17A are involved in both diseases, this study investigated their effects on the osteogenic differentiation of isolated human bone marrow-derived mesenchymal stem cells (hMSCs).

**Methods**: Differentiation of hMSCs into osteoblasts was induced in the presence or absence of IL-17A and/or TNF-α. Matrix mineralization (MM) was evaluated by alizarin red staining and alkaline phosphatase (ALP) activity. mRNA expression was measured by qRT-PCR for bone morphogenetic protein (BMP)-2 and Runx2, genes associated with osteogenesis, DKK-1, a negative regulator of osteogenesis, Schnurri-3 and receptor activator of nuclear factor kappa B ligand (RANKL), associated with the cross talk with osteoclasts, and TNF-α receptor type I and TNF-α receptor type II (TNFRII).

**Results:** TNF-α alone increased both MM and ALP activity. IL-17A alone increased ALP but not MM. Their combination was more potent. TNF-α alone increased BMP2 mRNA expression at 6 and 12 h. These levels decreased in combination with IL-17A at 6 h only. DKK-1 mRNA expression was inhibited by TNF-α and IL-17A either alone or combined. Supporting an imbalance toward osteoblastogenesis, RANKL expression was inhibited by TNF-α and IL-17A. However, TNF-α but not IL-17 alone decreased Runx2 mRNA expression at 6 h. In parallel, TNF-α but not IL-17 alone increased Schnurri-3 expression with a synergistic effect with their combination. This may be related to an increase of TNFRII overexpression.

**Conclusion:** IL-17 increased the effects of TNF-α on bone matrix formation by hMSCs. However, IL-17 decreased the TNF-α-induced BMP2 inhibition. Synergistic interactions between TNF-α and IL-17 were seen for RANKL inhibition and Schnurri-3 induction. Such increase of Schnurri-3 may in turn activate osteoclasts leading to bone destruction as in RA. Conversely, in the absence of osteoclasts, this could promote ectopic bone formation as in AS.

## Introduction

Interleukin-17A (IL-17A) is a pro-inflammatory cytokine that contributes to the pathogenesis of several inflammatory diseases (1, 2). In rheumatoid arthritis (RA), there is excessive bone destruction and defective bone repair leading to massive joint damage, while in ankylosing spondylitis (AS) there is excessive but ectopic ossification leading to syndesmophytes combined with systemic bone loss (1, 3–5). Moreover, increased levels of IL-17 in both diseases have suggested its contribution to these bone defects (4). Furthermore, IL-17A is considered as a new target for treatment of both RA and AS, as previously shown for TNF-α inhibition (6–11). Moreover, we have previously found that addition of inhibitors of IL-17A and TNF-α alone or even better in combination, decreases bone destruction in an *ex vivo* model of RA (12).

The mechanisms used by IL-17A to promote bone loss include activation of osteoclastogenesis, which occurs both directly and also through expression of receptor activator of nuclear factor kappa B ligand (RANKL) and M-CSF by stromal cells (4, 13). Moreover, IL-17A can induce target cells to produce pro-inflammatory cytokines such as IL-6, IL-1, CXCL8, TNF, and matrix metalloproteinases (2, 14–17). On the other hand, TNF-α inhibits osteoblastogenesis through increased expression of Dickkopf 1 (DKK-1) (18, 19) and induces bone loss by degradation of bone morphogenetic protein (BMP)-2 signaling via Smad ubiquitin regulatory factor (Smurf)1 and NF-kB (20–22). In contrast to their classical effects on bone loss, some studies have indicated that IL-17A and TNF-α possibly could increase osteogenesis (23–30). IL-17A can induce proliferation and differentiation of human mesenchymal stem cells (hMSCs) in a manner dependent on the generation of reactive oxygen species (ROS) (31). Moreover, IL-17A can significantly increased leptin production that inhibits adipogenesis and promotes osteogenesis on human bone marrow-derived mesenchymal stem cells (hMSCs) via JAK/STAT signaling (23). TNF-α can promote osteogenic differentiation through triggering NF-kB and enhancing the expression of BMP2 and RUNX2 (24, 25, 28).

Due to these conflicting results, our objective was to examine whether IL-17A alone and/or TNF-α, positively or negatively modulate osteogenic differentiation in hMSCs. To study these aspects, we focused on key genes involved in bone turnover: BMP2, Runx2, DKK-1, RANKL, and Schnurri-3, a gene recently associated with bone resorption in mice (32–34), but with a paucity of data on its role in the human context.

## Materials and Methods

### Cell culture, osteogenic induction, and experimental design

hMSCs were provided by the cell therapy department. They were obtained from residues of quality controls of bone marrow for transplantation harvested from adult donors after signing an informed consent. A fibroblast colony-forming unit (CFU-F) was used to optimize culture and expansion of hMSCs. Cells were cultured at 37°C in α-MEM (Lonza, Verviers, Belgium) supplemented with 10% fetal bovine serum (FBS-Hyclone, Thermo scientific, Saint Aubin, France), 2 mM l-glutamine, 100 U/ml penicillin, streptomycin. Cells were used between passage 3 and 6 at which cells were >99% stained negative for CD34 and CD45 and positive for CD73 and CD90 (antibodies obtained from PharMingen). For osteogenic differentiation, hMSCs were plated at a density of 5 × 10^3^ cells/cm^2^ and cultured in stem Xvivo Osteogenic/adipogenic base Medium (R&D systems, Lille, France), supplemented with 100 nM dexamethasone (Sigma, saint Quentin-Fallavier, France), 10 mM β-glycerophosphate (Sigma), and 50 μM ascorbic acid (Sigma). hMSCs were differentiated for 21 days in the absence or presence of 1 ng/ml TNF-α (R&D systems, Lille, France) and/or 50 ng/ml IL-17A (R&D systems) (23, 24, 31). Half of the medium was changed every 3 days.

### Mineralization assay

Cells were washed twice with PBS, fixed with 70% cooled ethanol for 1 h, and then washed with water. Cells were stained for 20 min at ambient temperature with alizarin red (pH: 4.2, 40 min, Sigma) and examined under light microscope. The red color obtained referred to calcium deposit.

### Alkaline phosphatase assay

hMSCs seeded in 12-well plates were lysed with the assay buffer (Abcam, Paris, France). The protein contents in the lysates were determined using the Bradford protein assay (Sigma). Ten microliters from the remaining lysate was mixed with 20 μl of MUP, used as a substrate (Abcam) in a 96-well plate, and incubated at room temperature for 30 min. Fluorescence intensity was measured at extension/emission of 360/440 nm. The alkaline phosphatase (ALP) activity was normalized to protein content and expressed as unit per microgram protein.

### Quantitative RT-PCR analysis

RNA was purified using RNeasy kits (Qiagen, Les Ulis, France). The concentration of RNA was quantified by spectrophotometry (SmartSpec™ 3000, Biorad, Hercules, CA, USA). Five hundred nanograms of total RNA was reverse transcribed with the Quanti Tec Reverse Transcription (Qiagen Kit) into cDNA. PCR amplification was performed on a Light Cycler (Roche Diagnostics, Switzerland) using Fast-Start™ DNA Master SYBR Green I real-time PCR kit (Roche Molecular Biochemicals, Switzerland). The expression of the genes was normalized to the expression of human cyclophilin B (CPB) (Qiagen; 5′tgtggtgtttggcaaagttc3′; 3′gtttatcccggctgtctgtc5′). The list of primers (Qiagen) is as follows: BMP2 (5′ccaccatgaagaatctttgga3′; 3′gagttggctgttgcaggttt5′), RUNX2 (5′gtggacgaggcaagagttt3′; 3′tggggtctgtaatctgactc5′), DKK-1 (5′ccttggatgggtattccaga3′; 3′tccatgagagccttttctcc5′), RANKL (5′accagcatcaaaatcccagg3′; 3′ccccaaagtatgttgcatcc5′), Shn 3 (5′ccctg agccataaccctgaa 3′; 3′gtaggacttggcgttggtgt 5′), TNF-α receptor type II (TNFRII) (5′ ggtctccttgctgctgtttc3′; 3′ccggagattctcaaatccaa5′), and TNF-α receptor type I (TNFRI) (5′ accaagtgccacaaaggaac 3′; 3′ctgcaattgaagcactggaa 5′).

### Statistical analysis

Analysis was performed using a Wilcoxon test from Graphpad Prism. *p*-Values were determined for every analysis. *p*-Values <0.05 were considered significant.

## Results

### Synergistic effects of IL-17A and TNF-α on extracellular matrix mineralization

**Figure 1:**
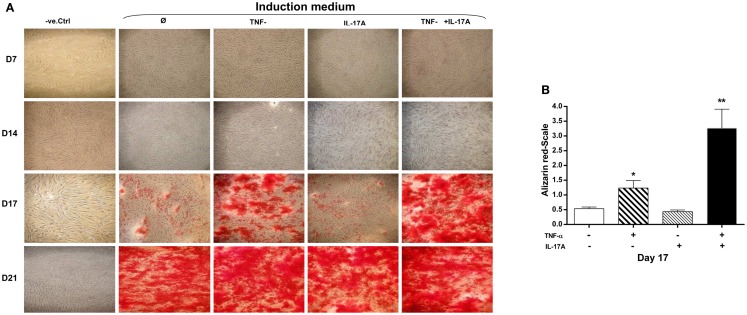
**Effects of IL-17A and/or TNF-α on extracellular matrix mineralization are shown**. hMSCs were plated at a density 5 × 10^3^ cell/cm^2^ and cultured for 21 days **(A)** in the absence (column 1) or presence (column 2) of osteogenic factors, 1 ng/ml TNF-α (column 3) or 50 ng/ml IL-17A (column 4), or both (column 5) were added or not to cultures. Plates were then stained with alizarin red, which colors calcium deposits in the extracellular matrix. **(B)** Results of day 17 were analyzed using the Wilcoxon test. *p  < 0.05; **p  < 0.005 vs. induction medium alone.

A key characteristic of MSCs is their ability to differentiate into cells of different tissue lineages. Mineralization of the extracellular matrix is a marker of hMSCs differentiation into osteoblasts. To evaluate the effects of IL-17A and/or TNF-α on MM, hMSCs were cultured for 21 days in a medium supplemented with osteogenic factors with and without cytokines. Alizarin red staining was used to visualize mineralization. As shown in Figure 1A, column 2, culture of MSC with osteogenic factors alone induced a weak MM, which appeared at day 17 and reached its maximum level at day 21. Addition of IL-17A alone did not modify this mineralization (Figure 1A, column 3; Figure 1B). Addition of TNF-α enhanced this mineralization at day 17 (Figure 1A, column 4; Figure 1B, *p  < 0.05), which was further enhanced in the presence of the two cytokines (Figure 1A, column 5; Figure 1B, **p  < 0.005). At day 21, maximum levels were observed in each condition except in the negative control. Thus, these results show that TNF-α but not IL-17A alone enhanced bone mineralization, which was further potentiated but without acceleration by IL-17A.

### Synergistic effects of IL-17A and TNF-α on alkaline phosphatase activity

**Figure 2:**
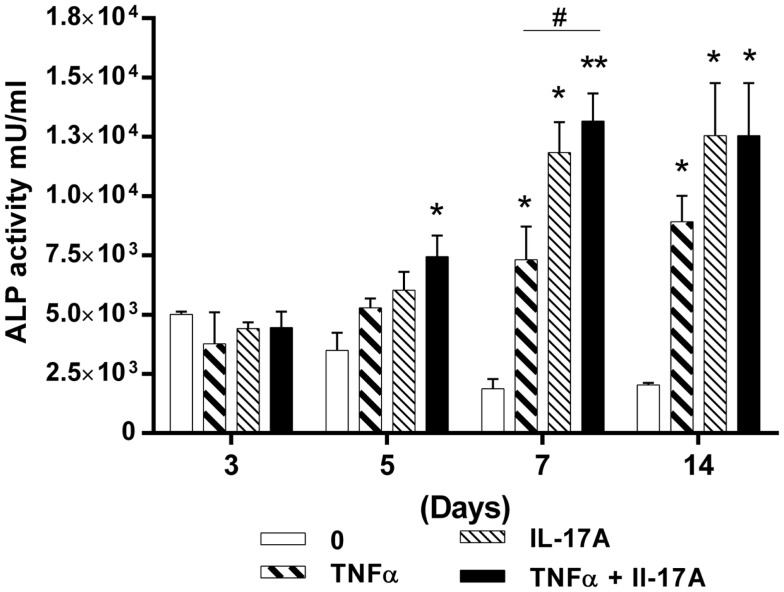
**Effects of IL-17A and/or TNF-α on alkaline phosphatase activity are shown**. hMSCs were plated at a density of 5 × 10^3^ cell/cm^2^, cells were cultured in osteogenic medium for 14 days, in the absence (0) or presence of TNF-α 1 ng/ml and/or IL-17A 50 ng/ml. ALP activity was measured by fluorometry. Results were analyzed using the Wilcoxon test. *p  < 0.05; **p  < 0.005 vs. induction medium alone.

Since TNF-α and IL-17A increased mineralization of the extracellular matrix, we next investigated their effects on ALP, an enzyme, which is essential for bone mineralization. ALP activity was measured at days 3, 5, 7, and 14 (Figure 2). At day 3, no difference in the presence or absence of cytokine was detected. Increased levels of ALP activity were detected at day 5 when the two cytokines were combined (4.4 × 10^3^ U/ml at day 3 vs. 7.4 × 10^3^ U/ml at day 5, *p  < 0.05) or at day 7 in the presence of each cytokine alone (Figure 2). Moreover, the combination of the two cytokines resulted in a further increase of ALP activity, the maximum level being achieved at day 7 (1.8 × 10^3^ U/ml without cytokine vs. 1.3 × 10^4^ U/ml with both cytokines, **p  < 0.005). Day 14 shows almost the same results as day 7.

Therefore, these results support those of the alizarin red staining, by showing that the combined action of IL-17A and TNF-α resulted in synergistically increasing and accelerating osteoblast differentiation from hMSCs. However, ALP activity appeared more sensitive to the effects of IL-17A alone.

### TNF-α but not IL-17A alone increases the expression levels of BMP2 mRNA

**Figure 3:**
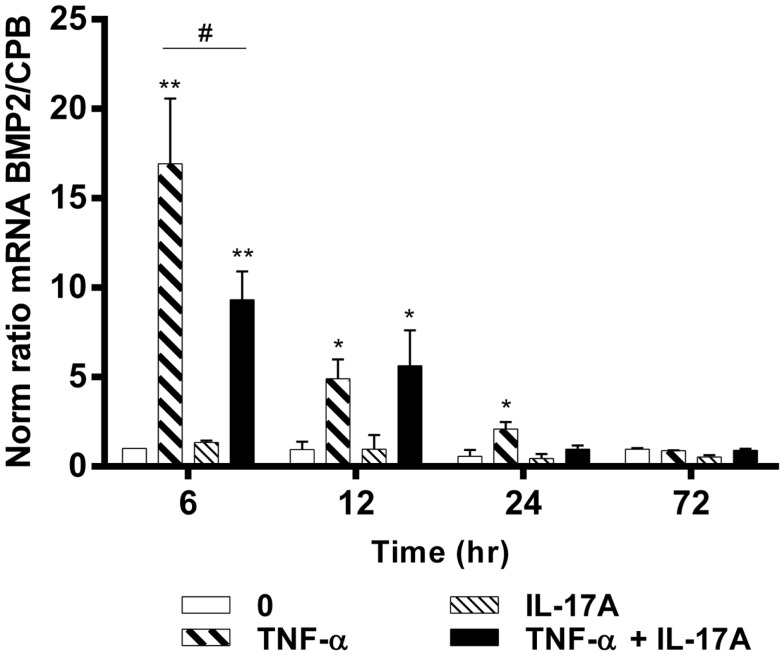
**Effects of IL-17A and TNF-α on BMP2 are shown**. hMSCs were cultured in osteogenic medium in the presence or absence of TNF-α 1 ng/ml and/or IL-17A 50 ng/ml. Osteogenic gene expression of BMP2 was measured by qRT-PCR at early time points of (6, 12, 24, and 72 h). Results were analyzed using the Wilcoxon test. *p  < 0.05; **p  < 0.005 vs. induction medium alone (0), ^#^*p * < 0.05 TNF-α alone vs. IL-17A + TNF-α.

To better understand the effects of IL-17A and TNF-α on the differentiation of hMSCs, mRNA expression levels of BMP2 were measured at 6, 12, 24, and 72 h. BMP2 plays an important role in the development of bone and cartilage and induces osteoblast differentiation in a variety of cell types (35). BMP2 mRNA expression was increased at 6 h with TNF-α alone (16-fold with TNF-α as compared with control without cytokine, **p  < 0.05), with a kinetic curve demonstrating a decrease upon time (Figure 3). IL-17A alone did not change BMP2 mRNA expression levels. The combination of the two cytokines resulted in a significant decrease of BMP2 mRNA expression as compared with the effects of TNF-α alone at 6 h (16-fold with TNF-α vs. 9-fold with TNF-α + IL-17A, ^#^*p * < 0.05). This inhibitory effect was not seen at 12 h (5.0-fold TNF-α vs. 5.4-fold IL-17A + TNF-α, NS). Overall, the combination of these two cytokines resulted in a significant increase of BMP2 mRNA expression as compared with controls (ninefold with TNF-α + IL-17A vs. control, *p  < 0.05, at 6 h, and 5.4-fold at 12 h). Therefore, these results showed that TNF-α increased the expression of BMP2 mRNA levels, an effect, which was inhibited by IL-17A.

### IL-17A and TNF-α increase the expression level of Shn3 mRNA but not those of Runx2

**Figure 4:**
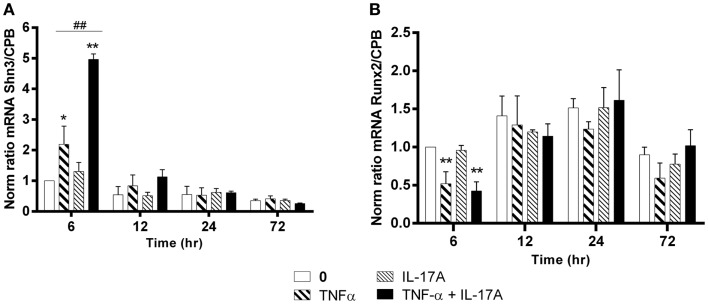
**Effects of Il-17A and TNF-α on Schnurri-3 and Runx2 are shown**. hMSCs were cultured in osteogenic medium in the presence or absence of TNF-α 1 ng/ml and/or IL-17A 50 ng/ml. Osteogenic gene expression Shn3 **(A)** and Runx2 **(B)** were measured by qRT-PCR at early time points of 6, 12, 24, and 72 h. Results were analyzed using the Wilcoxon test. *p  < 0.05; **p  < 0.005 vs. induction medium alone (0), ^#^*p * < 0.05 TNF-α alone vs. IL-17A + TNF-α.

Schnurri-3 (Shn3) is a zinc finger protein, which plays a key regulatory role in skeletal remodeling in mouse (36). In hMSCs, Shn3-mRNA expression levels increased significantly only at early 6 h in the presence of TNF-α alone (twofold with TNF-α, *p  < 0.05) with a kinetic curve demonstrating a decrease upon time. IL-17A alone did not change Shn3-mRNA expression levels (Figure 4A). The combination of the two cytokines resulted in a significant increase of Shn3-mRNA expression as compared with the effects of TNF-α alone at 6 h (fivefold with IL-17A + TNF-α vs. twofold with TNF-α, ^##^*p * < 0.005). No significant change of Shn3-mRNA expression levels was observed in the presence of either one or two cytokines between 12 and 24 h.

Runx2 is a key transcription factor that has been associated with osteogenesis (37). However, its mRNA expression levels showed no increase but rather unexpectedly a significant decrease at 6 h in the presence of TNF-α alone or combined to IL-17A as compared with control (0.5-fold with TNF-α or IL-17A+, TNF-α *P  < 0.05). No significant change of Runx2 mRNA expression levels was observed in the presence of either one or two cytokines between 12 and 72 h (Figure 4B).

Therefore, TNF-α alone and even more in combination with IL-17A increased Shn3-mRNA levels and decreased those of RUNX2 at early time points, while IL-17A alone showed no effects.

### IL-17A and TNF-α decrease RANKL and DKK-1 mRNA expression levels

**Figure 5:**
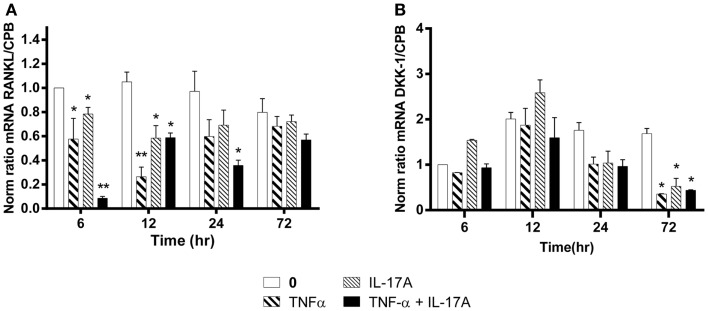
**Effects of Il-17A and TNF-α on RANKL and DKK-1 expression are shown**. hMSCs were cultured in osteogenic medium in the presence or absence of TNF-α 1 ng/ml and/or IL-17A 50 ng/ml. Osteogenic gene expression RANKL **(A)** and DKK-1 **(B)** were measured by qRT-PCR at early time points of 6, 12, and 24 h. Results were analyzed using the Wilcoxon test. *p  < 0.05; **p  < 0.005 vs. induction medium alone (0), ^#^*p * < 0.05 TNF-α alone vs. IL-17A + TNF-α.

The RANKL produced by osteoblasts plays a key role in osteoclast differentiation and activation (38). RANKL mRNA expression levels without cytokines remained stable over time, i.e., from 6 to 72 h (Figure 5A). In contrast, RANKL mRNA levels were significantly reduced as early as 6 h, when either IL-17A or TNF-α added alone (0.6-fold with TNF-α, 0.8-fold with IL-17A vs. 1-fold without cytokine, *p  < 0.05). The combined action of the two cytokines resulted in a more profound decrease of RANKL mRNA levels (0.1-fold with IL-17A combined to TNF-α vs. 1-fold without cytokine, **p  < 0.005). Moreover, this decrease was sustained upon time, since it was still observed at 24 h (0.35-fold with TNF-α combined to IL-17A vs. 1-fold without cytokine, *p  < 0.05). This result shows that these two cytokines might enhance osteogenesis by reducing RANKL expression.

Among the several molecules that negatively regulate Wnt signaling, DKK-1 prevents the activation of the Wnt signaling pathway (39). Upon differentiation of MSC into osteoblasts, DKK-1 mRNA expression levels without cytokines remained stable over time, i.e., from 12 to 72 h. In contrast, DKK-1 mRNA levels were significantly reduced at 72 h, when either IL-17A or TNF-α or both were added (0.3-fold with TNF-α, IL-17A, alone or combined vs. 1.6-fold without cytokine, *p  < 0.05) (Figure 5B). This suggests that these two cytokines might enhance osteogenesis by overcoming the negative modulation mediated by DKK-1.

Therefore, these results show a combined action of TNF-α and IL-17A on hMSCs increased osteogenesis through an inhibition of DKK-1 and RANKL gene expression.

### Combination of IL-17A and TNF-α increase TNFRII but not TNFRI expression

**Figure 6:**
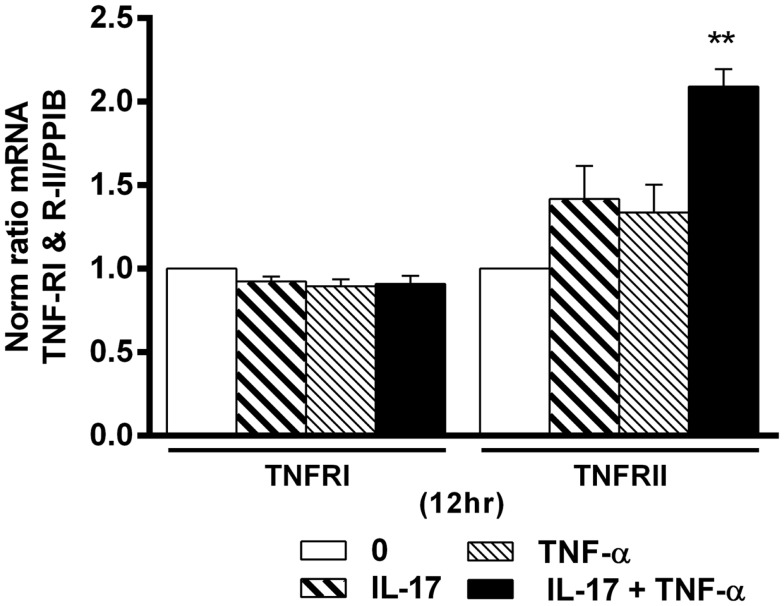
**Effects of Il-17A and TNF-α on TNFRI and TNFRII expression are shown**. hMSCs were cultured in osteogenic medium in the presence or absence of TNF-α 1 ng/ml and/or IL-17A 50 ng/ml. Gene expression of TNFRI and TNFRII were measured by qRT-PCR at 12 h. Results were analyzed using the Wilcoxon test. *p  < 0.005 vs. induction medium alone (0).

TNF-α receptor type I and TNFRII play an important role in cell proliferation, survival, and death (40, 41). Since IL-17 increased the effects of TNF-α in hMSCs, we looked at a possible effect on TNF-R expression. Addition of IL-17A and TNF-α alone or in combination had no effect on TNFRI mRNA expression levels (Figure 6). On the other hand, IL-17A and TNF-α alone had a very modest effect on TNFRII mRNA expression levels, but the combination induced a clear increase (2.0-fold with IL-17A + TNFα vs. 1-fold without cytokine at 12 h (*p  < 0.005). Therefore, these results show that the combined action of TNF-α and IL-17A on hMSCs may result from TNFRII but not TNFRI overexpression.

## Discussion

The goal of this study was to evaluate the effects of cytokines on the differentiation of human MSCs into bone forming cells, focusing on TNFα classical target for treatment and on IL-17, an emerging one, these two cytokines being known to interact. The first conclusion is the effect of TNF-α to promote osteogenic differentiation of hMSCs by increased deposition of calcium into the extracellular matrix, enhanced ALP activity, enhanced expression BMP2 mRNA levels, and decreased expression of RANKL and DKK-1 mRNA levels with an increase of Schnurri-3. As it is often the case, the effects of IL-17A alone were absent or limited in similar assays. The combination of TNF and IL-17 showed complex interactions with a net effect on enhanced matrix formation, ALP activity, and Schnurri-3 expression with reduced RANKL expression. A key surprising effect was the inhibition of the TNF-enhanced BMP2 expression when IL-17 was added.

Previous studies in human and murine systems have already shown an effect of IL-17A or TNF-α used alone on osteogenic induction (23–29, 31, 42). However, the classical view describes TNFα as an inhibitor of bone formation, more particularly with reference to RA. Among those studies, TNF-α has been reported to activate through TNF receptor-associated factor-2, a cascade of biochemical events involving NF-κB, AP-1, and MAPKs, leading to the activation of osteoclastic resorption and inhibition of osteoblast proliferation and matrix synthesis (43–45). Like TNF-α, IL-17A also activates NF-κB and AP-1 but through TRAF protein (46, 47). IL-17A inhibitory effect on matrix production in chondrocytes and osteoblasts leads to joint damage. It activates the production and function of MMPs, and a combination of IL-17A to TNF leads to irreversible cartilage damage in a murine model (3). IL-17A seems to increase ALP activity during osteogenesis and this is in harmony with two previous studies in the human system (23, 31).

While TNF-α was shown to increase BMP2 expression in different cell types like chondrocytes, endothelial cells, and dental pulp cells (48–50), little is known about the effects of TNF-α on BMP2 expression in hMSCs during the process of osteogenic differentiation. Here, TNF-α significantly increased BMP2 mRNA expression in hMSCs within 24 h of treatment (Figure 3) suggesting that TNF-α alone induces osteogenesis by increasing BMP2 expression. Much less is known on the effects of IL-17. IL-17A alone showed no effect. Instead of the expected potentiation with the combination with TNF, a reduction was observed at least at early time points.

Another factor involved in the regulation of osteogenesis is DKK-1, a major inhibitor of the Wnt signaling pathway (39). DKK-1 promotes internalization of the receptor complex and dampens the Wnt signal (51). This inhibition is potentiated in the presence of TNF-α (18, 19). However, our findings suggest that DKK-1 mRNA expression significantly decreased in the presence of TNF-α, which was associated with osteogenic induction. This discordance could be related to the use of MSCs, since opposite results have been obtained with synoviocytes (18). Moreover, serum levels of DKK-1 decreased following anti-TNFα therapy in RA patients (19) but not in AS patients (52). This suggests that the effects of TNFα on DKK-1 may not be direct, but may depend on factors associated with the disease status. Nevertheless, whatever the effect of TNF-α on DKK-1, we observed a strong correlation between the decrease of DKK-1 expression and the increase of bone mineralization. This observation is supported by Kowena et al. in a review on signaling (51).

Receptor activation of NF-κB ligand, a type II membrane protein of the TNF superfamily, is expressed in osteoblasts, osteocytes, and some activated T cells, specifically the Th17 cells (53, 54). RANKL is implicated in bone homeostasis, with TNFα known to induce its expression, and IL-17 having a modest effect. Additive effects of the combination TNFα + IL-17 on RANKL expression have been observed in human synoviocytes by us and others (55, 56). With hMSCs, opposite results were observed, since RANKL mRNA expression significantly decreased in the presence of IL-17A and/or TNF-α, which was associated with osteogenic induction.

Shn3 expression in mouse MSCs directly controls osteoblastic bone formation via Runx2, while indirectly regulating osteoclastic bone resorption (32, 33). Shn3-deficient mice show increased bone mass. However, there is a relative paucity of data on the role of Shn3 in the human context and its regulation with these cytokines has not been studied before. Our results showed that TNF-α alone or combined with IL-17A increased Shn3-mRNA expression levels at early time. Such increase would be a signal for osteoclast activation leading to bone destruction. This inter-cellular bridge would depend on whether osteoclasts are in close proximity with MSCs or osteoblasts. This difference could explain the different patterns of bone remodeling observed in RA and AS.

Since IL-17 increased the effects of TNF-α on hMSCs, we examined the possibility of an effect on TNF-R expression in order to explain the increased activity often observed with the combination of the two cytokines. TNFRI is ubiquitously expressed on nearly all cells but TNFRII has a more restricted expression, specifically on human lymphocytes (57–59) and MSCs (60, 61). Here, IL-17 and TNF alone or in combination had no effect on TNFRI expression. In contrast, TNFRII expression was regulated by the two cytokines in combination, with a modest not significant effect when used alone. These results are in line with those previously observed with synoviocytes, where a regulatory effect on TNFRII was also observed following exposure to TNF and IL-17. In this later case, however, IL-17 alone was able to increased TNFRII expression (62). Our results show that TNF-α and IL-17A may increase induce osteogenesis via TNFRII overexpression.

Our finding that TNFα and IL-17A activate osteogenesis, with IL-17A potentiating the effect of TNFα, may explain the mechanisms of ligament ossification with ectopic bone formation as observed in AS. It has been shown that the molecular pathways underlying AS recapitulate the presence of endochondral bone formation, where BMPs play a key role (63). Similar to their role in artery plaque formation during atherosclerosis, type II diabetes, and aortic valve disease, inflammatory cytokines are strongly suspected to induce ectopic bone formation (64–66). However, the conditions are different for the local and systemic bone destruction that is observed in RA. Both *in vitro* and *in vivo* results with TNF inhibitors are in line with the net effect of TNF on bone loss. Furthermore, the combined inhibition of TNF-α, IL-1, and IL-17 was more effective than the inhibition of a single cytokine to control inflammation and bone resorption in an *ex vivo* RA model (12).

Application to RA and AS pathogenesis and clinical manifestations indicates a key difference. In RA, the combination of TNF-α and IL-17 induces bone loss from massive osteoclast activation in sites, where osteoblasts and osteoclasts interact locally such as in juxta-articular sites (12). In contrast, AS is characterized by the ectopic bone formation from MSC derived from tendons and ligaments. At this particular site, osteoclasts are not directly presented and activated and the combination of TNF-α and IL-17 may then lead to ectopic ossification (1). Massive bone loss in whole bone such as vertebra and femoral neck is observed in AS as in RA.

In summary, this is the first study demonstrating that the two pro-inflammatory cytokines IL-17A and TNF-α can interact to induce osteogenic differentiation of human MSCs. Through complex interactions, IL-17A can potentiate the effects of TNF-α. An important new finding in the human context is the effect on Schunnri3, where the induction by TNF-α is further increased by IL-17A in a synergistic fashion. Such effect would provide an activation signal to osteoclasts. A close proximity to osteoclasts would be seen in whole bone as in juxta-articular sites of RA or vertebra in RA and AS. In tendons and ligaments where osteoclasts are not present, the lack of inter-cellular connection would lead to osteogenesis. At the site of tendon insertion to bone, the destructive pathway would be seen leading to erosion.

## Conflict of Interest Statement

The authors declare that the research was conducted in the absence of any commercial or financial relationships that could be construed as a potential conflict of interest.

## Abbreviations

AS, ankylosing spondylitis; BMP2, bone morphogenetic protein 2; DKK-1, dickkopf 1; MSC, mesenchymal stem cell; RA, rheumatoid arthritis; RANKL, receptor activator of nuclear factor kappa B ligand; Runx2, runt-related transcription factor-2; Shn3, Schnurri-3.

## References

[1] Miossec P IL-17 and Th17 cells in human inflammatory diseases. Microbes Infect (2009) 11:625–30 10.1016/j.micinf.2009.04.003 19371791

[2] Fossiez F Djossou O Chomarat P Flores-Romo L Ait-Yahia S Maat C T cell interleukin-17 induces stromal cells to produce proinflammatory and hematopoietic cytokines. J Exp Med (1996) 183:2593–603 10.1084/jem.183.6.2593 8676080 PMC2192621

[3] Koenders MI Marijnissen RJ Devesa I Lubberts E Joosten LA Roth J Tumor necrosis factor-interleukin-17 interplay induces S100A8, interleukin-1beta, and matrix metalloproteinases, and drives irreversible cartilage destruction in murine arthritis: rationale for combination treatment during arthritis. Arthritis Rheum (2011) 63:2329–39 10.1002/art.30418 21520013

[4] Kotake S Udagawa N Takahashi N Matsuzaki K Itoh K Ishiyama S IL-17 in synovial fluids from patients with rheumatoid arthritis is a potent stimulator of osteoclastogenesis. J Clin Invest (1999) 103:1345–52 10.1172/JCI5703 10225978 PMC408356

[5] Lubberts E Joosten LA van de Loo FA Schwarzenberger P Kolls J van den Berg WB Overexpression of IL-17 in the knee joint of collagen type II immunized mice promotes collagen arthritis and aggravates joint destruction. Inflamm Res (2002) 51:102–4 10.1007/BF02684010 11930902

[6] Miossec P Kolls JK Targeting IL-17 and T(H)17 cells in chronic inflammation. Nat Rev Drug Discov (2012) 11:763–76 10.1038/nrd3794 23023676

[7] Baeten D Sieper J Emery P Braun J van der Heijde D McInnes I The anti-IL17A monoclonal antibody secukinumab (AIN457) showed good safety and efficacy in the treatment of active ankylosing spondylitis. Arthritis Rheum (2010) 62:3840 Abstract L7.

[8] Koenders MI Kolls JK Oppers-Walgreen B van den Bersselaar L Joosten LA Schurr JR Interleukin-17 receptor deficiency results in impaired synovial expression of interleukin-1 and matrix metalloproteinases 3, 9, and 13 and prevents cartilage destruction during chronic reactivated streptococcal cell wall-induced arthritis. Arthritis Rheum (2005) 52:3239–47 10.1002/art.21342 16200598

[9] Koenders MI Lubberts E Oppers-Walgreen B van den Bersselaar L Helsen MM Di Padova FE Blocking of interleukin-17 during reactivation of experimental arthritis prevents joint inflammation and bone erosion by decreasing RANKL and interleukin-1. Am J Pathol (2005) 167:141–9 10.1016/S0002-9440(10)62961-6 15972960 PMC1603454

[10] Appel H Maier R Wu P Scheer R Hempfing A Kayser R Analysis of IL-17(+) cells in facet joints of patients with spondyloarthritis suggests that the innate immune pathway might be of greater relevance than the Th17-mediated adaptive immune response. Arthritis Res Ther (2011) 13:R95 10.1186/ar3370 21689402 PMC3218910

[11] Genovese MC Van den Bosch F Roberson SA Bojin S Biagini IM Ryan P LY2439821, a humanized anti-interleukin-17 monoclonal antibody, in the treatment of patients with rheumatoid arthritis: a phase I randomized, double-blind, placebo-controlled, proof-of-concept study. Arthritis Rheum (2010) 62:929–39 10.1002/art.27334 20131262

[12] Chabaud M Miossec P The combination of tumor necrosis factor alpha blockade with interleukin-1 and interleukin-17 blockade is more effective for controlling synovial inflammation and bone resorption in an ex vivo model. Arthritis Rheum (2001) 44:1293–303 10.1002/1529-0131(200106)44:6<1293::AID-ART221>3.0.CO;2-T 11407688

[13] Sato K Suematsu A Okamoto K Yamaguchi A Morishita Y Kadono Y Th17 functions as an osteoclastogenic helper T cell subset that links T cell activation and bone destruction. J Exp Med (2006) 203:2673–82 10.1084/jem.20061775 17088434 PMC2118166

[14] Li X Yuan FL Lu WG Zhao YQ Li CW Li JP The role of interleukin-17 in mediating joint destruction in rheumatoid arthritis. Biochem Biophys Res Commun (2010) 397:131–5 10.1016/j.bbrc.2010.05.111 20513356

[15] Chabaud M Page G Miossec P Enhancing effect of IL-1, IL-17, and TNF-alpha on macrophage inflammatory protein-3alpha production in rheumatoid arthritis: regulation by soluble receptors and Th2 cytokines. J Immunol (2001) 167:6015–20 10.4049/jimmunol.167.10.6015 11698482

[16] Hwang SY Kim JY Kim KW Park MK Moon Y Kim WU IL-17 induces production of IL-6 and IL-8 in rheumatoid arthritis synovial fibroblasts via NF-kappaB- and PI3-kinase/Akt-dependent pathways. Arthritis Res Ther (2004) 6:R120–8 10.1186/ar1038 15059275 PMC400429

[17] Koshy PJ Henderson N Logan C Life PF Cawston TE Rowan AD Interleukin 17 induces cartilage collagen breakdown: novel synergistic effects in combination with proinflammatory cytokines. Ann Rheum Dis (2002) 61:704–13 10.1136/ard.61.8.704 12117676 PMC1754191

[18] Diarra D Stolina M Polzer K Zwerina J Ominsky MS Dwyer D Dickkopf-1 is a master regulator of joint remodeling. Nat Med (2007) 13:156–63 10.1038/nm1538 17237793

[19] Wang SY Liu YY Ye H Guo JP Li R Liu X Circulating Dickkopf-1 is correlated with bone erosion and inflammation in rheumatoid arthritis. J Rheumatol (2011) 38:821–7 10.3899/jrheum.100089 21362762

[20] Guo R Yamashita M Zhang Q Zhou Q Chen D Reynolds DG Ubiquitin ligase Smurf1 mediates tumor necrosis factor-induced systemic bone loss by promoting proteasomal degradation of bone morphogenetic signaling proteins. J Biol Chem (2008) 283:23084–92 10.1074/jbc.M709848200 18567580 PMC2517001

[21] Kaneki H Guo R Chen D Yao Z Schwarz EM Zhang YE Tumor necrosis factor promotes Runx2 degradation through up-regulation of Smurf1 and Smurf2 in osteoblasts. J Biol Chem (2006) 281:4326–33 10.1074/jbc.M509430200 16373342 PMC2647592

[22] Krum SA Chang J Miranda-Carboni G Wang CY Novel functions for NFkappaB: inhibition of bone formation. Nat Rev Rheumatol (2010) 6:607–11 10.1038/nrrheum.2010.133 20703218 PMC3078572

[23] Noh M Interleukin-17A increases leptin production in human bone marrow mesenchymal stem cells. Biochem Pharmacol (2012) 83:661–70 10.1016/j.bcp.2011.12.010 22197587

[24] Huang H Zhao N Xu X Xu Y Li S Zhang J Dose-specific effects of tumor necrosis factor alpha on osteogenic differentiation of mesenchymal stem cells. Cell Prolif (2011) 44:420–7 10.1111/j.1365-2184.2011.00769.x 21951285 PMC6495272

[25] Hess K Ushmorov A Fiedler J Brenner RE Wirth T TNFalpha promotes osteogenic differentiation of human mesenchymal stem cells by triggering the NF-kappaB signaling pathway. Bone (2009) 45:367–76 10.1016/j.bone.2009.04.252 19414075

[26] Mountziaris PM Tzouanas SN Mikos AG Dose effect of tumor necrosis factor-alpha on in vitro osteogenic differentiation of mesenchymal stem cells on biodegradable polymeric microfiber scaffolds. Biomaterials (2010) 31:1666–75 10.1016/j.biomaterials.2009.11.058 19963268 PMC2813987

[27] Yu RY Zeng BJ Liu YS Zhou YS Recombinant human tumor necrosis factor-alpha promotes human adipose-derived stromal cells transforming into osteoblast in vitro. Beijing Da Xue Xue Bao (2012) 44:475–80 22692324

[28] Lu Z Wang G Dunstan CR Zreiqat H Short-term exposure to tumor necrosis factor-alpha enables human osteoblasts to direct adipose tissue-derived mesenchymal stem cells into osteogenic differentiation. Stem Cells Dev (2012) 21:2420–9 10.1089/scd.2011.0589 22296271

[29] Glass GE Chan JK Freidin A Feldmann M Horwood NJ Nanchahal J TNF-alpha promotes fracture repair by augmenting the recruitment and differentiation of muscle-derived stromal cells. Proc Natl Acad Sci U S A (2011) 108:1585–90 10.1073/pnas.1018501108 21209334 PMC3029750

[30] Osta B Benedetti G Miossec P Classical and paradoxical effects of TNF-alpha on bone homeostasis. Front Immunol (2014) 5:48 10.3389/fimmu.2014.00048 24592264 PMC3923157

[31] Huang H Kim HJ Chang EJ Lee ZH Hwang SJ Kim HM IL-17 stimulates the proliferation and differentiation of human mesenchymal stem cells: implications for bone remodeling. Cell Death Differ (2009) 16:1332–43 10.1038/cdd.2009.74 19543237

[32] Wein MN Jones DC Shim JH Aliprantis AO Sulyanto R Lazarevic V Control of bone resorption in mice by Schnurri-3. Proc Natl Acad Sci U S A (2012) 109:8173–8 10.1073/pnas.1205848109 22573816 PMC3361406

[33] Jones DC Wein MN Oukka M Hofstaetter JG Glimcher MJ Glimcher LH Regulation of adult bone mass by the zinc finger adapter protein Schnurri-3. Science (2006) 312:1223–7 10.1126/science.1126313 16728642

[34] Jones DC Wein MN Glimcher LH Schnurri-3 is an essential regulator of osteoblast function and adult bone mass. Ann Rheum Dis (2007) 66(Suppl 3):iii49–51 10.1136/ard.2007.078352 17934096 PMC2095286

[35] Kamiya N Mishina Y New insights on the roles of BMP signaling in bone-A review of recent mouse genetic studies. Biofactors (2011) 37:75–82 10.1002/biof.139 21488130 PMC3551451

[36] Jones DC Wein MN Glimcher LH Schnurri-3: a key regulator of postnatal skeletal remodeling. Adv Exp Med Biol (2007) 602:1–13 10.1007/978-0-387-72009-8_1 17966382

[37] Ducy P Zhang R Geoffroy V Ridall AL Karsenty G Osf2/Cbfa1: a transcriptional activator of osteoblast differentiation. Cell (1997) 89:747–54 10.1016/S0092-8674(00)80257-3 9182762

[38] Suda T Takahashi N Udagawa N Jimi E Gillespie MT Martin TJ Modulation of osteoclast differentiation and function by the new members of the tumor necrosis factor receptor and ligand families. Endocr Rev (1999) 20:345–57 10.1210/edrv.20.3.0367 10368775

[39] Li J Sarosi I Cattley RC Pretorius J Asuncion F Grisanti M Dkk1-mediated inhibition of Wnt signaling in bone results in osteopenia. Bone (2006) 39:754–66 10.1016/j.bone.2006.03.017 16730481

[40] Faustman DL Davis M TNF receptor 2 and disease: autoimmunity and regenerative medicine. Front Immunol (2013) 4:478 10.3389/fimmu.2013.00478 24391650 PMC3870411

[41] Faustman D Davis M TNF receptor 2 pathway: drug target for autoimmune diseases. Nat Rev Drug Discov (2010) 9:482–93 10.1038/nrd3030 20489699

[42] Mojsilovic S Krstic A Ilic V Okic-Dordevic I Kocic J Trivanovic D IL-17 and FGF signaling involved in mouse mesenchymal stem cell proliferation. Cell Tissue Res (2011) 346:305–16 10.1007/s00441-011-1284-5 22160457

[43] Kelliher MA Grimm S Ishida Y Kuo F Stanger BZ Leder P The death domain kinase RIP mediates the TNF-induced NF-kappaB signal. Immunity (1998) 8:297–303 10.1016/S1074-7613(00)80535-X 9529147

[44] Granet C Miossec P Combination of the pro-inflammatory cytokines IL-1, TNF-alpha and IL-17 leads to enhanced expression and additional recruitment of AP-1 family members, Egr-1 and NF-kappaB in osteoblast-like cells. Cytokine (2004) 26:169–77 10.1016/j.cyto.2004.03.002 15149634

[45] Zhao L Huang J Zhang H Wang Y Matesic LE Takahata M Tumor necrosis factor inhibits mesenchymal stem cell differentiation into osteoblasts via the ubiquitin E3 ligase Wwp1. Stem Cells (2011) 29:1601–10 10.1002/stem.703 21809421 PMC3708970

[46] Kehlen A Thiele K Riemann D Langner J Expression, modulation and signalling of IL-17 receptor in fibroblast-like synoviocytes of patients with rheumatoid arthritis. Clin Exp Immunol (2002) 127:539–46 10.1046/j.1365-2249.2002.01782.x 11966773 PMC1906300

[47] Shalom-Barak T Quach J Lotz M Interleukin-17-induced gene expression in articular chondrocytes is associated with activation of mitogen-activated protein kinases and NF-kappaB. J Biol Chem (1998) 273:27467–73 10.1074/jbc.273.42.27467 9765276

[48] Csiszar A Smith KE Koller A Kaley G Edwards JG Ungvari Z Regulation of bone morphogenetic protein-2 expression in endothelial cells: role of nuclear factor-kappaB activation by tumor necrosis factor-alpha, H2O2, and high intravascular pressure. Circulation (2005) 111:2364–72 10.1161/01.CIR.0000164201.40634.1D 15851600

[49] Fukui N Zhu Y Maloney WJ Clohisy J Sandell LJ Stimulation of BMP-2 expression by pro-inflammatory cytokines IL-1 and TNF-alpha in normal and osteoarthritic chondrocytes. J Bone Joint Surg Am (2003) 85-A(Suppl 3):59–66 12925611 10.2106/00004623-200300003-00011

[50] Okabe T Matsushima K Regulation of ALP activity by TNF-alpha on human dental pulp. J Endod (2006) 32:516–20 10.1016/j.joen.2005.12.007 16728240

[51] Kawano Y Kypta R Secreted antagonists of the Wnt signalling pathway. J Cell Sci (2003) 116:2627–34 10.1242/jcs.00623 12775774

[52] Kwon SR Lim MJ Suh CH Park SG Hong YS Yoon BY Dickkopf-1 level is lower in patients with ankylosing spondylitis than in healthy people and is not influenced by anti-tumor necrosis factor therapy. Rheumatol Int (2012) 32:2523–7 10.1007/s00296-011-1981-0 21833531

[53] Kong YY Feige U Sarosi I Bolon B Tafuri A Morony S Activated T cells regulate bone loss and joint destruction in adjuvant arthritis through osteoprotegerin ligand. Nature (1999) 402:304–9 10.1038/46303 10580503

[54] Nakashima T Hayashi M Fukunaga T Kurata K Oh-Hora M Feng JQ Evidence for osteocyte regulation of bone homeostasis through RANKL expression. Nat Med (2011) 17:1231–4 10.1038/nm.2452 21909105

[55] Page G Miossec P RANK and RANKL expression as markers of dendritic cell-T cell interactions in paired samples of rheumatoid synovium and lymph nodes. Arthritis Rheum (2005) 52:2307–12 10.1002/art.21211 16052586

[56] Tunyogi-Csapo M Kis-Toth K Radacs M Farkas B Jacobs JJ Finnegan A Cytokine-controlled RANKL and osteoprotegerin expression by human and mouse synovial fibroblasts: fibroblast-mediated pathologic bone resorption. Arthritis Rheum (2008) 58:2397–408 10.1002/art.23653 18668542

[57] Ware CF Crowe PD Vanarsdale TL Andrews JL Grayson MH Jerzy R Tumor necrosis factor (TNF) receptor expression in T lymphocytes. Differential regulation of the type I TNF receptor during activation of resting and effector T cells. J Immunol (1991) 147:4229–38 1661312

[58] Arnett HA Mason J Marino M Suzuki K Matsushima GK Ting JP TNF alpha promotes proliferation of oligodendrocyte progenitors and remyelination. Nat Neurosci (2001) 4:1116–22 10.1038/nn738 11600888

[59] Irwin MW Mak S Mann DL Qu R Penninger JM Yan A Tissue expression and immunolocalization of tumor necrosis factor-alpha in postinfarction dysfunctional myocardium. Circulation (1999) 99:1492–8 10.1161/01.CIR.99.11.1492 10086975

[60] Bocker W Docheva D Prall WC Egea V Pappou E Rossmann O IKK-2 is required for TNF-alpha-induced invasion and proliferation of human mesenchymal stem cells. J Mol Med (Berl) (2008) 86:1183–92 10.1007/s00109-008-0378-3 18600306

[61] Pimentel-Muinos FX Seed B Regulated commitment of TNF receptor signaling: a molecular switch for death or activation. Immunity (1999) 11:783–93 10.1016/S1074-7613(00)80152-1 10626900

[62] Zrioual S Ecochard R Tournadre A Lenief V Cazalis MA Miossec P Genome-wide comparison between IL-17A- and IL-17F-induced effects in human rheumatoid arthritis synoviocytes. J Immunol (2009) 182:3112–20 10.4049/jimmunol.0801967 19234208

[63] Carter S Braem K Lories RJ The role of bone morphogenetic proteins in ankylosing spondylitis. Ther Adv Musculoskelet Dis (2012) 4:293–9 10.1177/1759720X12444175 22859928 PMC3403253

[64] Demer LL Vascular calcification and osteoporosis: inflammatory responses to oxidized lipids. Int J Epidemiol (2002) 31:737–41 10.1093/ije/31.4.737 12177011

[65] Doherty TM Asotra K Fitzpatrick LA Qiao JH Wilkin DJ Detrano RC Calcification in atherosclerosis: bone biology and chronic inflammation at the arterial crossroads. Proc Natl Acad Sci U S A (2003) 100:11201–6 10.1073/pnas.1932554100 14500910 PMC208734

[66] Helske S Kupari M Lindstedt KA Kovanen PT Aortic valve stenosis: an active atheroinflammatory process. Curr Opin Lipidol (2007) 18:483–91 10.1097/MOL.0b013e3282a66099 17885417

